# The Role of Perceived School Diversity Climate in the School Involvement of Ethnically and Racially Minoritized Parents in Germany and the United Kingdom

**DOI:** 10.1002/jcop.70069

**Published:** 2025-12-07

**Authors:** Alison E. F. Benbow, Gülseli Baysu, Priscilla Krachum Ott, Maja K. Schachner, Aileen Edele

**Affiliations:** ^1^ Humboldt University Berlin Germany; ^2^ Queen's University Belfast UK; ^3^ Martin Luther University Halle‐Wittenberg Halle‐Wittenberg Germany

**Keywords:** comparative research, equality, diversity and inclusion, institutional practices, parental school involvement, race/ethnicity, school diversity climate

## Abstract

This cross‐sectional online survey considered how school diversity climate and parental involvement are related for ethnically or racially minoritized and majoritized parents of school‐aged children (*N* = 1074) in the United Kingdom and Germany. Cross‐group comparisons showed that parents in the United Kingdom reported more multicultural and assimilative, and less discriminatory school climates than parents in Germany. Across countries, minoritized parents reported less multiculturalism, more assimilationism, and more school discrimination than majoritized parents. As expected, multicultural school climate was positively related, and discriminatory school climate was negatively related to parental school involvement. Unexpectedly, assimilationism was positively related to school involvement for minoritized parents in Germany and not related to school involvement in the UK sample. Overall, our findings indicate that parental perceptions of school conditions matter for their school involvement. Consequently, schools may benefit from evaluating, refining, and communicating their diversity approaches to increase parental involvement, promote school adaptation, and reduce educational inequities.

Parental involvement has long been considered an important means of increasing educational achievement of ethnically and racially minoritized pupils and consequently decreasing educational disparities between these pupils and majoritized groups (e.g., Hill 2022; Wild 2021). However, much of the literature around family‐school cooperation has focused on familial conditions that may foster or hinder parental involvement, while neglecting conditions that are inherent in schools and school policies. This approach has favored explanations of differences in involvement between ethnically and racially minoritized and majoritized (hereafter minoritized and majoritized) parents^1^ that downplay the importance of intergroup relations within the school context (e.g., racism and/or perceptions of disparate cultural values; Wild 2021) and systemic inequity and exclusion (Kollender 2024). To address this imbalance, the current study examined the relationship between parental perceptions of school diversity climate and their self‐reported school involvement cross‐comparatively with minoritized and majoritized parents in two national contexts. Specifically, it considered how national context differences (such as equality, diversity, and inclusion policies) and the differing societal and school realities of being part of a minoritized or majoritized group related to perceptions of school diversity climate, and how school diversity climate related to parents' self‐reported school involvement.

## 1 Parental School Involvement: Definition, Measurement, Relationships to Child Outcomes

Socialization and education are central tasks of parenting that take up a substantial amount of family life with far‐reaching consequences for child development and adaptation (Hill 2022). Parents encourage children's social, emotional, and academic growth (Green et al. 2007) and help them to understand and adopt the goals, beliefs, and practices of a given society (Hill 2021). Ideally, schools are an important resource and source of support for parents in this endeavor, but this depends on the extent to which successful family‐school cooperation can be established. Educational researchers and policymakers increasingly highlight active parental involvement in educational processes as a critical factor in children's and adolescents' academic success and as beneficial to their school‐based relationships with peers and teachers (e.g., Desforges and Abouchaar 2003; Jeynes 2012; Wild 2021). However, the perception and understanding of what constitutes effective parental involvement—and what roles and activities are desirable and appropriate for parents in this context—often differ widely between parents and schools (e.g., Antony‐Newman 2018; Myers 2015). In particular, parents' perspectives on and expectations regarding family‐school cooperation are currently under‐researched (Hill 2022).

To date, the research focus has been on how to actively engage parents in different types of school involvement (e.g., Epstein 2010). Unfortunately, this research is marked by a broad and sometimes inconsistent use, measurement, and application of the concept of parental involvement, adding complexity without increasing clarity (Boonk et al. 2018). Studies have considered home‐based parental involvement, referring to a range of actions taken at home to support children's learning, and/or school‐based parental involvement, describing parents' participation and decision making in contact with the school, such as communicating with teachers, taking part in meetings, events, and volunteering (e.g., Fan and Chen 2001). In addition, they have included both behavioral and/or psychological aspects (sometimes also referred to as parental engagement: their emotional involvement in and commitment to education) of parental involvement (Boonk et al. 2018).

Meta‐analyses following this research tradition generally confirm positive relationships (with small to medium effect sizes) of parental involvement on both behavioral and psychological aspects of home‐based and school‐based forms of involvement with students' achievement and behavioral outcomes, but also highlight studies that find negative or no relationships between these variables (e.g., Castro et al. 2015; Fan and Chen 2001; Jeynes 2003, 2007; Patall et al. 2008). These meta‐analyses and other research syntheses also point to significant relations of parental class, race, gender, immigrant status, and age of child with different types of parental involvement and children's outcomes. For instance, while both home‐based and school‐based involvement have been found to be relevant to students' achievement motivation and achievement (Boonk et al. 2018; Fan and Chen 2001; Kim et al. 2020), there is some evidence that school‐based involvement may relate to student achievement more strongly for minoritized than majoritized families, while home‐based involvement may be more strongly related to achievement for majoritized than minoritized families (Boonk et al. 2018). The reasons for the differential effects of school involvement in minoritized and majoritized families are not clearly known; one potential explanation is that school‐based involvement is more visible to the school and teachers, and that this, together with the contact opportunities inherent in school‐based involvement, may help to counter stereotypical notions about minoritized families.

Differences in the school involvement of families are currently often addressed by applying an individualistic, motivational lens that highlights the family as the “source” of successful involvement. That is, differences in parents' role construction, sense of efficacy, and perceptions of life‐context variables (i.e., parents' perceptions of their knowledge and skills, and their personal time and energy for involvement; Walker et al. 2005) are considered as the main factors for differences in levels of parental involvement (e.g., Epstein et al. 2018). In contrast, explanations that are inherent in school practices or policies are largely neglected (except with regard to involvement invitations, see e.g., Yulianti et al. 2020). To address this gap, this study examines the relationship between the school diversity climate as perceived by the parents and their school involvement. Specifically, we focused on school‐based parental involvement because school‐based parental involvement should be more closely related to school context conditions, whereas home‐based parental involvement may be more independent of them.

## 2 Parental School Involvement of Minoritized Families

Ethnically and racially minoritized families are frequently identified in the extant literature as families who would particularly benefit from close family‐school cooperation, based on the assumption that this would help to reduce continuing educational disparities between ethnically and racially minoritized students and their majoritized peers (e.g., OECD 2023). A recent meta‐analysis by Kim et al. (2020) provides a case in point: they summarized 14 quantitative studies with regard to the effects of parental influence indicators (i.e., psychological engagement, behavioral involvement, and SES/educational level) on immigrant students' achievement‐related motivation. The authors report that both psychological engagement and behavioral involvement positively relate to first‐ and second‐generation immigrant students' achievement motivation, irrespective of parental education and SES. They suggest, as others do, that increasing parental involvement and engagement is therefore a fruitful avenue for the reduction of educational disparities.

At the same time as ethnic and racially minoritized families are tasked with this expectation, they are also perceived as facing particular challenges regarding their readiness for school involvement, despite their high aspirations and motivations for their children's education (e.g., Crosnoe and Ansari 2015; Kim et al. 2020) and despite evidence that they are actively steering the quality of the education opportunities afforded to their children in the face of adversity (e.g., Aral and Juang 2025; Yurdakul and Altay 2022). These challenges are often framed in terms of familial factors such as a lack of financial resources, of language skills, and/or of knowledge (about parenting, child development, about teaching), or an unwillingness to connect with schools (factors that are also applied to other families perceived as disadvantaged, such as those with low SES; Antony‐Newman 2018; Doucet 2011; Isik‐Ercan 2018).

These explanations that encapsulate a deficit view on parental involvement stand in contrast to the growing body of—often qualitative—literature highlighting that barriers for minoritized parents stem from mismatches between parental and school expectations about school involvement. These mismatches relate to types of involvement and ways of communicating (Antony‐Newman 2018), to school practices that are steeped in (hidden) cultural values and rituals that hinder interaction (Wild 2021) excluding minoritized parents from school access (Turney and Kao 2009) or devaluing their involvement (Sohn and Wang 2006). Furthermore, many minoritized families experience discrimination and racism in their interactions with schools, further discouraging their involvement (e.g., Wild 2021).

Barriers to parental involvement of minoritized families are therefore likely to stem from institutional conditions in school and school policy based on stereotypical and uniform perceptions of minoritized families and systemic inequity, as well as from familial conditions. In consequence, schools may fail to take advantage of minoritized parents as an important resource and source of knowledge regarding their child and relevant cultural practices and activities that can increase inclusive education (Antony‐Newman 2018) to the detriment of all children in culturally diverse schools. However, as many of these arguments are theoretical in nature or based on qualitative evidence, research that is more generalizable is needed to better understand the role of institutional conditions in encouraging or discouraging involvement of minoritized parents. In light of these gaps in knowledge, such research should explicitly focus on the parental perspective on intergroup relations in the school and classroom and their own school involvement. Previous studies suggest that school diversity climate could be an important factor in the school involvement of minoritized parents.

## 3 School Climate Research

Within schools, intergroup relations are guided by (often implicit) ideological assumptions about the importance of cultural identities, group hierarchies, and the need to address inequality and discrimination, so‐called diversity approaches (e.g., Whitley and Webster 2019). In some schools, minoritized groups are expected to adopt the values and behaviors of a (presumed) ‘majority’ culture (i.e., assimilationism), in others, the benefits of cultural diversity are acknowledged, and heritage culture and intercultural learning are fostered (i.e., multiculturalism). Other approaches include so‐called “color‐blind” [sic] stances, approaches based on optimal intergroup‐contact conditions, critical consciousness approaches, and polyculturalism, to name just a few (see Bardach et al. 2024 and Schachner et al. 2021 for more detailed descriptions of these approaches). The distinction between these approaches has not always been clear‐cut in terms of the definition and operationalization of constructs, however, and some (e.g., multiculturalism) have been researched more than others (e.g., polyculturalism; Bardach et al. 2024). In addition to diversity approaches, research in this area has also considered the extent to which the school climate is experienced as discriminatory (e.g., Baysu et al. 2023), which, while not a diversity approach, is nonetheless an important marker of the school climate and how the institutions are handling diversity.

While it is unlikely that a school exclusively adheres to one specific approach in practice, different school diversity approaches are evident in school policy statements (Celeste et al. 2019), teacher beliefs and self‐reported behavior (Schotte et al. 2021; Schwarzenthal et al. 2023), and student perceptions (Schachner et al. 2016). The different school diversity approaches relate in a complex fashion to varied consequences for students' school adjustment. For instance, school belonging and educational achievement were lower when pupils perceived school diversity climates as discriminatory or assimilationist and higher when they perceived multicultural climates (Baysu et al. 2023; Schachner et al. 2016). At the same time, no connection was found between diversity‐related teacher beliefs and student achievement (Schotte et al. 2021). A recent meta‐analysis (Bardach et al. 2024) indicates that a multicultural climate is significantly and positively related to intergroup attitudes, achievement, motivation, and belonging, albeit with mostly small effect sizes. In a departure from other research, assimilationism was not assessed as a separate approach in this study, but subsumed under the so‐called “color‐blind” approaches, which showed no overall relationship to intergroup, academic, or socio‐emotional outcomes. The meta‐analysis further highlights the complexity of the current field of research, finding that the person reporting (teacher vs. student), the level of schooling (primary vs. secondary), and the percentage of majority students present in the classroom affected some of the relationships between school climate approaches and outcomes. The question of who benefits (most) from schools' diversity approaches also remains open: While some studies indicate that the positive effects of diversity approaches are similar for majoritized and minoritized students (e.g., in Germany: Schachner et al. 2019), other research suggests the positive effects might be stronger for the minoritized group (e.g., in Belgium: Celeste et al. 2019). Relating to teachers, there is some evidence that multicultural approaches are more beneficial for teachers than assimilationist approaches (e.g., Schwarzenthal et al. 2023; Ulbricht et al. 2022).

Nonetheless, the approaches addressed in this literature should also tie in with institutional practices involving minoritized parents: for example, it is likely that an assimilationist approach would exclude parental knowledge about relevant cultural practices and activities, their experiences, and ways of supporting their children, while a multicultural approach would entail an openness to including this knowledge. Similarly, schools that are perceived as discriminatory towards minoritized groups should elicit different parental involvement responses than those that are not: for instance, parents may withdraw from these contexts or, conversely, increase their efforts to address racism and support/protect their children.

Though general school climate is known to shape the relationships between schools and parents (Wang and Degol 2016), minoritized parents' perspectives on school diversity climates remain under‐researched. As a notable exception, a recent study in the US considered parental perceptions of school relationships and their links to bullying/victimization (Fu et al. 2024). The authors found that parental perceptions of a school's culture and inclusiveness differed by ethnic group, with Black and bi‐ or multiracial parents perceiving the least school inclusiveness of all ethnic groups. Furthermore, youth victimization was negatively associated with perceptions of the school's culture of inclusiveness and equity more strongly in minoritized parents than in White parents. For Black, Asian, and bi‐/multiracial parents, youth victimization was also more negatively associated with perceptions of the school's interest in reaching out and engaging parents than for other parents. While this was not the focus of their research, these results suggest that minoritized parents differ in their perceptions of school diversity climate and, when faced with the victimization of their children, do not feel that their schools are doing enough to create a school climate that fosters equity and inclusion or encourages parental school involvement. In light of these findings and prior research on school diversity climate and on parental involvement, we believe a focus on parents' perceptions of school diversity climate may help to shed light on institutional practices that are related to parents' school involvement and have previously been neglected in the literature.

## 4 Current Study

The current study aimed to address the following gaps in the literature: first, we wanted to provide insights into parents' perceptions of their own school involvement, which we defined as parental involvement in school (as opposed to home‐based involvement). Following Boonk et al. (2018), we apply a broad conceptualization of school‐based parental involvement, including parental involvement behaviors (such as attending meetings, providing materials, and volunteering), and psychological/emotional aspects of school involvement (specifically, whether they feel welcomed and included, their endorsement of the school, and their relationship to the child's teachers). Second, we wanted to overcome the biased focus on familial conditions in parental school involvement and examine the association of institutional and contextual practices with parental involvement. Third, we wanted to provide a view of parental involvement that disentangles processes related to the societal and school realities faced by ethnically and racially minoritized parents from other factors such as parental efficacy, socioeconomic status, or national context effects. We addressed these gaps by taking a cross‐comparative approach to the relationship between parental perceptions of school diversity climate and their self‐reported school‐based school involvement. Our levels of comparison are two‐fold: first, we compare ethnically and racially minoritized parents from two national contexts, Germany and the UK. Second, we compare ethnically and racially minoritized versus majoritized parents across these two national contexts.

While similar in many respects (e.g., both are multicultural, Western European, democratic societies with strong economies and similar quality of life; World Data Info 2025), the United Kingdom and Germany differ in the type of migration they have experienced since the Second World War (predominantly (post)colonial and, more recently, EU work migration to the United Kingdom vs. repeated waves of work and refugee migration to Germany). The countries also differ in terms of their national approach and dedication to multiculturalism policies and the implementation of equality, diversity, and inclusion (EDI) policies in educational contexts. As of 2020, Germany scored 3 of 8 points on the Multiculturalism Policy Index (2025) across several areas, including Affirmation, School Curriculum, Media, Dress Code Exemptions, Dual Citizenship, Funding, Bilingual Education, and Affirmative Action, while the UK scored 6 of 8 points.

Furthermore, in Germany, policies regarding education are mainly federally regulated, and commitment to and spread of EDI policies, though indicated by overarching national antidiscrimination laws, vary greatly by federal state (e.g., The Standing Conference of the Ministers of Education and Cultural Affairs of the Länder in the Federal Republic of Germany 2017). Consequently, clear and binding guidelines for schools are lacking, and management of diversity lies largely within the responsibility of individual principals and teaching staff. In contrast, all schools in the United Kingdom are required to define EDI goals and document how they are reaching their targets by the nationwide Equality Act of 2010 (e.g., Department for Education 2014). Assuming that decisive management of diversity should lead to a better and more rigorous implementation of EDI policies, we expected that parents in the United Kingdom would perceive schools to be less assimilationist, less discriminatory, and provide more opportunities for cultural learning than parents in Germany (H1a, H1b, and H1c). We chose multicultural, assimilationist, and discriminatory, because these three dimensions of school diversity climate are well researched with regard to school practices (e.g., Gomolla and Kollender 2022¸ Krachum Ott et al. 2025; Phalet and Baysu 2020) and other school adaptation outcomes (see above review of past research).

As stated above, our second set of comparisons focused on ethnically and racially minoritized versus majoritized parents across the two countries and thus speaks to experiences that are common for minoritized parents irrespective of their national context. As mentioned, past research names a multitude of factors that may relate to parental school involvement, including parental class, gender, age of child, and self‐efficacy, as well as race and immigrant status. Given the likely heterogeneity within the two groups we created to reflect social positionality as minoritized or majoritized, we expected that some of the observed differences in the school‐based involvement between minoritized parents and majoritized parents should be explained by differences in some of these factors, notably SES and self‐efficacy, which are consistently named in the literature. For instance, differences in their experiences of and acceptance within the school system might provide majoritized parents with more self‐efficacy for school involvement than minoritized parents. Similarly, lower SES is related to less involvement and might be confounded with membership in the minoritized group for some participants (Green et al. 2007).

More primarily, however, we expected minoritized parents to have different perceptions regarding the school's diversity climate. This is because minoritized families face different societal and school realities than majoritized families, including children experiencing more in‐school ethnic‐racial discrimination (e.g., Moffitt et al. 2018) and stereotyping (e.g., Glock et al. 2013), lower teacher expectations (Lorenz et al. 2016), and disproportionate representation in lower‐tier tracks or schools (e.g., Kemper 2015) to name just a few. This is also evidenced in the steps taken by minoritized parents to socialize and prepare their children for racism (Umaña‐Taylor and Hill 2020). By comparison, White parents tend to be underprepared for conversations about race and racism, to employ evasive strategies that deny or are indifferent to racial inequality, or prepare their children to maintain privilege (e.g., Kaiser et al. 2025; Nieri et al. 2024). Given these differences, we expected minoritized parents to perceive schools as providing fewer opportunities for cultural learning, practicing more assimilationism, and having a more discriminatory climate than majoritized parents (H2a, H2b, and H2c).

Finally, we wanted to clarify the relationship between school diversity climate and parental involvement for ethnically and racially minoritized parents. In line with the multi‐faceted nature of school‐based involvement and to gain a broader picture of the relationships between school diversity climate and parental school involvement, we considered parental self‐reports regarding their involvement and volunteering behaviors, their relationships to teachers, their general endorsement of their child's school and their perceptions of the school's invitations to participate (Walker et al. 2005). Based on previous findings that highlight the benefits of multicultural approaches such as cultural learning and the detrimental effects of assimilationist and discriminatory practices in schools (e.g., Baysu et al. 2023; Schachner et al. 2016), we expected lower parental involvement when minoritized parents perceived a discriminatory or assimilationist school climate and higher parental involvement when they perceived a climate in which cultural learning takes place irrespective of the national context (H3a, b, c). Given the limited research examining how school diversity climate relates to parental involvement, we explored how diversity climate is associated with different dimensions of parental involvement. In addition, we considered these relationships in majoritized parents. This made it possible to disentangle potential effects of national context from minoritized status, whereby similarities in minoritized parents, but not majoritized parents in both countries would point to status effects, while similarities between minoritized parents and majoritized parents in one country, but not the other would speak to national context effects, and similarities between all four groups would indicate a more general process. However, considering the ambiguity regarding the effects of diversity approaches for minoritized and majoritized students discussed above, we did not have specific expectations as to how the relationships between perceived diversity climate and parental school involvement would look for majoritized parents. To determine the unique relationship between perceived school diversity climate and parental involvement, we controlled for parental SES and efficacy, as these are well‐established familial predictors of parental school involvement that could distort the findings.

## 5 Methods

### 5.1 Author Positionality

The first author, a white woman, immigrated to Germany as a child with her family. She has first‐hand experience of the challenges of immigrant families negotiating a school system, both as a pupil and as a parent of children of color. Several authors also have lived experience of migration and of discrimination/racism, some are mothers of school‐age children, and these experiences partially drive our interest in these research questions.

### 5.2 Participants and Procedure

Our sample was comprised of 1074 parent participants (*M*_age_ = 40, SD = 9; 64% female, 36% male, zero nonbinary, three non‐respondents) whose children were attending primary (51%) and secondary (49%) schools and were 10.63 years old on average (SD = 3.62; 48% female, 52% male, 0 nonbinary, 8 non‐respondents). Table 1 displays their demographics overall and separately by national context.

**Table 1:** Distributions of sample characteristics: Overall, by country and ethnically and racially minoritized/majoritized status.

		Overall			UK			Germany		
		Total	Min	Maj	Total	Min	Maj	Total	Min	Maj
Cases	*n* (%)	1074 (100)	537 (50)	537 (50)	470 (44)	224 (48)	246 (52)	604 (56)	313 (52)	291 (48)
Child's school: Primary (vs. secondary)	*n* (%)	552 (51)	295 (55)	257 (48)	265 (56)	135 (60)	130 (53)	287 (48)	160 (51)	127 (44)
Gender: Female (vs. male)^a^	*n* (%)	689 (64)	365 (68)	324 (60)	219 (47)	147 (66)	197 (80)	298 (49)	218 (70)	127 (44)
Country of residence (UK/Germany)										
…is place of birth	*n* (%)	809 (75)	273 (51)	536 (100)	347 (74)	123 (55)	246 (100)	462 (77)	172 (55)	290 (100)
…is child's place of birth	*n* (%)	1002 (93)	474 (88)	528 (98)	430 (92)	188 (84)	242 (98)	572 (95)	286 (91)	286 (98)
…same first language or bilingual	*n* (%)	921 (86)	384 (72)	537 (100)	406 (86)	160 (71)	246 (100)	354 (59)	224 (79)	289 (99)
Religion										
No affiliation	*n* (%)	387 (36)	115 (21)	272 (51)	204 (43)	58 (26)	146 (59)	183 (30)	57 (18)	126 (43)
Christian	*n* (%)	478 (45)	224 (42)	254 (47)	188 (40)	88 (39)	100 (41)	290 (48)	136 (44)	154 (53)
Muslim	*n* (%)	156 (14)	149 (28)	7 (1)	51 (11)	51 (23)	0	105 (17)	98 (31)	7 (2)
Other	*n* (%)	53 (5)	49 (9)	4 (1)	27 (6)	27 (12)	0	26 (4)	22 (7)	4 (1)
Age	*M* (SD)	40.01 (8.92)	38.16 (8.14)	41.87 (9.28)	39.83 (7.48)	39.30 (6.90)	40.32 (7.96)	40.15 (9.87)	37.37 (8.83)	43.13 (10.10)
Child's age	*M* (SD)	10.63 (3.62)	10.29 (3.65)	10.97 (3.55)	9.99 (3.57)	9.68 (3.58)	10.28 (3.58)	11.13 (3.56)	10.73 (3.65)	11.55 (3.42)

^a^ Nobody chose the nonbinary response option for gender.

Overall, the samples were quite similar across national contexts and minoritized/majoritized subsamples with regard to, for instance, the proportion of parents of primary and secondary school children, and the average child's age. The proportion of minoritized parents and their children born in the country of residence, of English/German as a first language or bilingual language, and the proportion of Christians were similar for minoritized parents in the United Kingdom and Germany. However, the minoritized groups in the United Kingdom and Germany did differ in terms of their ethnicity, consistent with the countries differing historical migration populations discussed above: in the UK subsample, 64% self‐identified as Asian or Black, compared to about one in five of the German subsample.^2^ Other notable differences include the fact that more majoritized parents reported no religious affiliation than minoritized parents (51% vs. 21%) and that less than 1% of the majoritized group reported being Muslim or belonging to another faith (compared to 28% and 9% for the minoritized groups). Majoritized parents were slightly older on average than minoritized parents (42 vs. 38 years, respectively), though this was most pronounced in the German sample (43 vs. 37 years, respectively). Finally, there was a higher percentage of females in the majoritized group in the United Kingdom (80%) and a lower percentage of females in the majoritized group in Germany (44%) than in the minoritized groups (UK: 66%; Germany: 70%). Participants were recruited in 2021 via online market research survey providers Prolific (UK) and Bilendi (Germany). We pre‐screened available samples to identify majoritized and minoritized parents of school children. We defined ethnic and racially minoritized parents based on four questions: first language non‐English/non‐German, or non‐White race/ethnicity, or parents and/or self not born in the UK/Germany, or a religious minority (such as Muslim or Hindu). Ethnic and racially majoritized parents were those who were White, parents and self‐born in the UK/Germany, first language English/German. Participants were fully informed of the purpose of the study and their rights as participants of psychological research; they were remunerated for their participation following the usual standards of the market research providers used. Participants answered a questionnaire about their impressions of the diversity of their child's school, their practices/preparation for dealing with diversity, and their school involvement. To ensure the quality of responses, attention checks were included in the questionnaire and used to screen out participants from the final sample (*n*_UK_ = 45; *n*_Germany_ = 39).

### 5.3 Measures

All measures were based on established scales and presented in the national language of each context (English and German). Unless otherwise indicated, we created scales by averaging across the corresponding items, with higher scores indicating higher levels on the relevant dimension. We provide example items in this section, and a full list of items can be viewed on the Open Science Framework (OSF; https://osf.io/j568k/overview).

#### 5.3.1 Parental School Involvement

Parents completed the Parent–Teacher Involvement Questionnaire (Walker et al. 2005), which includes four subscales of parental school involvement relating to behavioral as well as psychological aspects of parental involvement with school. Items were all rated on five‐point Likert‐type scales (1 *completely disagree* to 5 *completely agree*): *Parent's Endorsement of the Child's School* was assessed with four statements (e.g., “My child's school is a good place for my child to be.”; Cronbach's α = 0.91). The *Quality of the Relationship between Parent and Teacher* was assessed with four statements (e.g., “I feel comfortable talking with my child's teacher about my child.”; Cronbach's α = 0.87). *General School Invitations* were assessed with four statements (e.g., “This school lets me know about meetings and special school events.”; Cronbach's α = 0.78). *Parent's Involvement and Volunteering at School* was assessed with three statements (e.g., “Whenever possible I volunteer at my child's school.”; Cronbach's α = 0.56). Measurement invariance was established using a multigroup confirmatory factor analysis (MGCFA) approach. As reported in Table 2, metric invariance was established, but scalar invariance was only partial and was only achieved by freeing the intercept of Item 3 of the involvement and volunteering at school scale (“Whenever possible, I volunteer at my child's school”). Therefore, interpretations including this item across the two examined national contexts and minoritized/majoritized groups should be considered with caution.

**Table 2:** Measurement invariance for key study variables.

	Model fit						Model comparison			
	χ^2^	df	CFI	TLI	SRMR	RMSEA [90% CI]	Models	Δχ^2^ (Δdf)	ΔCFI	ΔRMSEA
Parental involvement										
Configural (M1)	856.862	336	0.943	0.928	0.065	0.076 [0.070, 0.083]				
Metric (M2)	952.422	369	0.936	0.927	0.090	0.077 [0.071, 0.083]	M2‐M1	95.560 (33)***	0.007	0.001
Scalar (M3)	1160.587	402	0.917	0.913	0.105	0.084 [0.078, 0.090]	M3‐M2	208.165 (33)***	−0.019	0.007
Partial scalar (M3a)^a^	1093.343	399	0.924	0.920	0.104	0.081 [0.075, 0.086]	M3a‐M2	140.921 (30)***	−0.012	0.004
School diversity climate										
Configural (M1)	213.624	128	0.974	0.963	0.046	0.050 [0.038, 0.062]				
Metric (M2)	235.060	149	0.973	0.968	0.056	0.047 [0.035, 0.058]	M2‐M1	21.436 (21)	−0.001	0.003
Scalar (M3)	357.312	170	0.942	0.939	0.068	0.064 [0.055, 0.074]	M3‐M2	122.253 (21)***	−0.031	0.012
Partial scalar (M3a)^b^	287.958	167	0.963	0.960	0.062	0.052 [0.042, 0.062]	M3a‐M2	52.899 (18)***	−0.010	0.005

Abbreviations: Δ, change in the parameter; CFI, Comparative Fit Index; CI, confidence interval; df, degrees of freedom; RMSEA, Root Mean Square Error of Approximation; SRMR, Standardized Root Mean Square Residual; TLI, Tucker‐Lewis Index; χ^2^, chi‐square.

^a^ In this model, the intercept of item Assimilationism 3 was unconstrained.

^b^ In this model, the intercept of item Parental Involvement and Volunteering in School 3 was unconstrained.

*** *p* < 0.001.

#### 5.3.2 Parents' Perception of the School Diversity Climate

Parents were asked to rate statements about how the school manages diversity on five‐point Likert scales (1 *completely disagree* to 5 *completely agree*) adapted from previous research (e.g., Baysu et al. 2023; Schachner et al. 2016). To represent a *Multicultural Climate* pupils' *heritage culture and intercultural learning* were assessed with four items (e.g., “Pupils learn about the countries where the families of other students came from.”; Cronbach's α = 0.85). *Assimilationist Climate* was assessed with three items (e.g., “Pupils are expected to follow what is customary in the UK/Germany.”; Cronbach's α = 0.63). *School Discriminatory Climate* was assessed with three items (e.g., “Pupils with a different background are excluded or bullied more than others.” Cronbach's α = 0.80). Measurement invariance was established using MGCFA. As reported in Table 2, metric invariance was established, but scalar invariance was only partial and was achieved by freeing the intercepts of Item 3 of the assimilationism scale (“Teachers want only English to be spoken in school”). Therefore, interpretations including this item across the two examined national contexts and minoritized/majoritized groups should be considered with caution.

#### 5.3.3 Demographic Information and Control Variables

Parents reported their age, gender, nationality, first language, and religion, as well as their children's age and level of school (primary/secondary). They rated their *socioeconomic status* (*SES)* by marking their position on a ladder representing where people stand in the UK/Germany (scaled from 1 *worst off* to 10 *best off*). They also self‐reported on their perceived parental efficacy regarding school decisions, services, and communication (Ball 2014) on a five‐point scale (1 *completely disagree* to 5 *agree entirely*). The scale comprised eight items (e.g., “When faced with a problem involving my child in school, I decide what to do and then do it.”; Cronbach's α = 0.85).

## 6 Results

### 6.1 Missing Data, Descriptives, and Preliminary Analyses

Data were missing in less than 1% of cases in our study variables (with no more than two missing cases on any items) and were randomly distributed. Analyses in SPSS (Version 29), therefore, applied listwise deletion of missing values, while analyses in MPlus (Version 8) applied Full Information Maximum Likelihood as the standard to replace missing values. Data, Syntax, and Outputs for all analyses can be viewed on the OSF (https://osf.io/j568k/overview). Means, standard deviations, and correlations of our key variables are displayed in Table 3. We explored potential differences in our study control and dependent variables in a 2 (context: Germany/UK) x 2 (minoritized/majoritized status) between‐subjects MANOVA in SPSS with SES, parental efficacy, and the four parental school involvement variables as dependent variables. This showed that the combined dependent variables were significantly affected by context, *V* = 0.148, *F*(6, 1065) = 30.93, *p* < 0.001, η_p_² = 0.148, and by minoritized/majoritized status, *V* = 0.018, *F*(6, 1065) = 3.24, *p* = 0.004, η_p_² = 0.018. There were no interactions between context and status. Follow‐up univariate ANOVAs indicated main effects of context and minoritized/majoritized status on SES and parental efficacy, *F*s(1, 1070) ≥ 6.56, *p*s ≤ 0.011, η_p_²s ≥ 0.006. Parents in Germany reported higher SES, but less parental efficacy than parents in the United Kingdom (means and standard deviations for these and other group comparisons are provided in Table 3). Across contexts, minoritized parents reported higher SES and more efficacy than majoritized parents.^3^ With regard to parental school involvement, follow‐up univariate ANOVAs indicated main effects of context on all four variables, *F*s(1, 1070) ≥ 2.97, *p*s ≤ 0.06, η_p_²s ≥ 0.003, with parents reporting more involvement in the United Kingdom than in Germany. We found no main effects of minoritized/majoritized status on parental school involvement, except for on parents' involvement and volunteering, *F*(1, 1070) = 4.93, *p* = 0.027, η_p_²s = 0.005, where minoritized parents reported more involvement and volunteering than majority parents.

**Table 3:** Intercorrelations, reliabilities, means, and standard deviations for main study variables (overall and by group and context).

	Correlations								Total			United Kingdom						Germany					
												Min			Maj			Min			Maj		
	2	3	4	5	6	7	8	9	α	*M*	SD	α	*M*	SD	α	*M*	SD	Α	*M*	SD	α	*M*	SD
Variables																							
1 SES	0.05	0.03	0.04	0.06*	0.11***	0.15***	0.16***	0.16***	—	6.03	1.68	—	5.83	1.45	—	5.45	1.44	—	6.40	1.82	—	6.27	1.71
2 EFF	—	0.19***	0.24***	0.07*	0.24***	0.37***	0.34***	0.48***	0.85	3.77	0.70	0.84	3.90	0.69	0.84	3.81	0.68	0.84	3.78	0.67	0.87	3.64	0.72
3 MUL		—	0.02	−0.30***	0.49***	0.44***	0.45***	0.18***	0.85	3.84	0.79	0.82	3.97	0.75	0.83	4.04	0.71	0.86	3.62	0.86	0.86	3.81	0.73
4 ASS			—	0.32***	0.12***	0.09*	0.13***	0.13***	0.59	3.70	0.73	0.54	3.86	0.64	0.57	3.66	0.67	0.65	3.75	0.86	0.63	3.56	0.72
5 SDC				—	−0.29***	−0.24***	−0.26***	0.08**	0.80	2.51	1.00	0.79	2.36	0.97	0.65	2.00	0.79	0.79	2.95	1.00	0.81	2.57	0.95
6 END					—	0.76***	0.74***	0.33***	0.91	4.05	0.81	0.90	4.27	0.67	0.91	4.30	0.72	0.88	3.86	0.83	0.93	3.89	0.86
7 PTRQ						—	0.75***	0.40***	0.87	3.95	0.80	0.83	4.12	0.66	0.86	4.13	0.77	0.89	3.81	0.85	0.88	3.81	0.80
8 GSI							—	0.39***	0.78	4.06	0.74	0.76	4.24	0.67	0.76	4.16	0.71	0.79	3.95	0.77	0.81	3.97	0.74
9 IAV								—	0.56	3.42	0.87	0.55	3.39	0.87	0.49	3.34	0.84	0.62	3.56	0.88	0.62	3.37	0.89

*Note: N* = 1074. Correlations are Pearson's *r*.

Abbreviations: ASS, assimilationism; EFF, self‐efficacy; END, endorsement; GSI, general school invitations; IAV, parental involvement and volunteering in school; Maj, ethnically and racially majoritized parents; Min, ethnically and racially minoritized parents; MUL, multiculturalism; PTRQ, parent‐teacher relationship quality; SDC, school discriminatory climate; SES, socioeconomic status.

* *p* < 0.05

** *p* < 0.01

*** *p* < 0.001.

### 6.2 Comparative Perceptions of School Diversity Climate

To address our research hypothesis regarding national context differences (H1a, H1b, and H1c) and differences between minoritized and majoritized parents (H2a, H2b, and H2c) in perceived school diversity climate, we conducted a 2 (context: Germany/UK) x 2 (minoritized/majorized status) between‐subjects MANCOVA in SPSS with the three school climate variables as dependent variables controlling for SES and parental efficacy as covariates. Results showed that the combined dependent variables were significantly affected by context, *V* = 0.12, *F*(3, 1066) = 48.40, *p* < 0.001, η_p_² = 0.12, and minoritized/majoritized status, *V* = 0.04, *F*(3, 1066) = 6.95, *p* < 0.001, η_p_² = 0.04. There were no interactions between context and status. Follow‐up univariate ANCOVAs indicated that there were significant differences in parental perception of cultural learning and discrimination in the United Kingdom and in Germany, *F*s(1, 1068) ≥ 35.04, *p*s ≤ 0.001, η_p_²s ≥ 0.032 (see Table 3 for means across groups). As expected, parents from the United Kingdom perceived more cultural learning and less discrimination in their schools than parents in Germany (supporting H1a and H1c). However, and counter to our expectations, they also rated assimilationism higher in schools than German parents did (falsifying H1b). The same ANCOVAs also highlighted differences in the perceptions of school diversity management of minoritized and majoritized parents, *F*s(1, 1068) ≥ 6.02, *p*s ≤ 0.001, η_p_²s ≥ 0.011. As expected, minoritized parents reported less cultural learning, more assimilationism, and more discrimination in schools than majoritized parents, thereby supporting H2a, H2b, and H2c.

### 6.3 The Relationship Between Perceptions of School Diversity Climate and Parental School Involvement

To address our third hypothesis regarding the relationships between perceptions of school diversity climate and parental school involvement, we conducted multigroup structural equation modeling (MGSEM) in MPlus, regressing the parental school involvement variables onto the school climate variables and controlling for SES and self‐efficacy. Tables 3, 4, 5 details the fit indices for the fully constrained model, the freely estimated model, and the model that showed the best fit to the data. To address the question of whether processes are more general, status‐specific, or country‐specific, we compared constraining pathways to be equal across all four groups, or equal in the minority and majority groups, or equal within countries, respectively. In our final solution (summarized in Table 5), all regression paths to SES and self‐efficacy were freely estimated in each group, all paths regressing volunteering onto the school climate variables, and all paths that were regressed onto assimilationism were freely estimated in the German subsamples only, and all other paths were constrained to be equal.

**Table 4:** Multigroup structural equation models comparing the regression of parental involvement variables on school diversity climate variables.

	Model fit								Model comparisons			
	χ^2^	df	CFI	TLI	SRMR	RMSEA [90% CI]	AIC	BIC	Models	Δχ^2^ (Δdf)	ΔAIC	ΔBIC
Fully constrained model (M1)	3957.096***	2175	0.895	0.892	0.087	0.055 [0.053, 0.058]	84448.278	86106.334				
Freely estimated model (M2)	3872.659***	2115	0.897	0.890	0.079	0.056 [0.053, 0.058]	84483.841	86440.645	M1‐M2	84.437 (60)*	−35.563	−334.311
Final solution (M3)	3881.815***	2142	0.898	0.893	0.081	0.055 [0.052, 0.058]	84438.997	86261.365	M1‐M3	75.281 (33)***	9.281	−155.031
									M2‐M3	9.156 (27)	44.844	179.280

*Note:* In these models, the intercepts of item Assimilationism 3 and item Parental Involvement and Volunteering in School 3 were unconstrained.

Abbreviations: Δ, change in the parameter; AIC, Akaike Information Criterion; BIC, Bayesian Information Criterion; CFI, Comparative Fit Index; CI, confidence interval; df, degrees of freedom; RMSEA, root mean square error of approximation; SRMR, standardized root mean square residual; TLI, Tucker–Lewis Index; χ^2^, chi‐square.

* *p* < 0 .05

*** *p* < 0.001.

**Table 5:** Standardized parameter estimates of the relationships between school diversity climate and parental school involvement.

	UK								Germany							
	IAV		END		PTRQ		GSI		IAV		END		PTRQ		GSI	
	Maj	Min	Maj	Min	Maj	Min	Maj	Min	Maj	Min	Maj	Min	Maj	Min	Maj	Min
	*β* (SE)	*β* (SE)	*β* (SE)	*β* (SE)	*β* (SE)	*β* (SE)	*β* (SE)	*β* (SE)	*β* (SE)	*β* (SE)	*β* (SE)	*β* (SE)	*β* (SE)	*β* (SE)	*β* (SE)	*β* (SE)
SES	**0.11 (0.08)**	**0.06 (0.07)**	**0.06 (0.06)**	**0.14** * **(0.06)**	**0.06 (0.06)**	**0.19** ** **(0.06)**	**0.08 (0.06)**	**0.17** *** **(0.06)**	**0.24** *** **(0.06)**	**0.10 (0.06)**	**0.14** ** **(0.05)**	**0.16** ** **(0.05)**	**0.18** *** **(0.04)**	**0.18** *** **(0.04)**	**0.17** *** **(0.04)**	**0.19** *** **(0.04)**
EFF	**0.55** *** **(0.08)**	**0.67** *** **(0.07)**	**−0.01 (0.07)**	**0.12** * **(0.06)**	**0.24** *** **(0.07)**	**0.34** *** **(0.06)**	**0.16** * **(0.07)**	**0.28** *** **(0.06)**	**0.61** *** **(0.07)**	**0.71** *** **(0.07)**	**0.21** *** **(0.05)**	**0.20** *** **(0.06)**	**0.44** *** **(0.05)**	**0.40** *** **(0.04)**	**0.33** * **(0.05)**	**0.32** *** **(0.05)**
ASS	−0.04 (0.09)	−0.04 (0.08)	0.07 (0.06)	0.07 (0.06)	0.01 (0.06)	0.01 (0.06)	0.04 (0.07)	0.04 (0.06)	**−0.02 (0.10)**	**−0.21 (0.13)**	**0.15** * **(0.08)**	**0.34** *** **(0.08)**	**−0.02 (0.07)**	**0.19** ** **(0.07)**	**0.09 (0.08)**	**0.35** *** **(0.08)**
SDC	0.01 (0.04)	0.01 (0.10)	−0.25*** (0.05)	−0.33*** (0.05)	−0.18*** (0.04)	−0.27*** (0.06)	−0.27*** (0.05)	−0.35*** (0.05)	**−0.26** *** **(0.09)**	**0.30** * **(12)**	−0.26*** (0.05)	−0.31*** (0.05)	−0.22*** (0.05)	−0.23*** (0.05)	−0.32*** (0.05)	−0.35*** (0.06)
MUL	0.20** (0.04)	0.19* (0.08)	0.40*** (0.04)	0.42*** (0.04)	0.31*** (0.04)	0.35*** (0.04)	0.36*** (0.04)	0.37*** (0.04)	**0.34** *** **(0.07)**	**0.07 (0.07)**	0.34*** (0.03)	0.44*** (0.04)	0.29*** (0.03)	0.34*** (0.04)	0.34*** (0.04)	0.41*** (0.04)

*Note:* In these models, the intercepts of item Assimilationism 3, item Parental Involvement and Volunteering in School 3, and items Self‐Efficacy 3, 6, and 7 were unconstrained. Freely estimated paths are marked in bold.

Abbreviations: ASS, assimilationism; EFF, self‐efficacy; END, endorsement; GSI, general school invitations; IAV, parental involvement and volunteering in school; Maj, ethnically and racially majoritized parents; Min, ethnically and racially minoritized parents; MUL, multiculturalism; PTRQ,parent‐teacher relationship quality; SDC, School Discriminatory Climate; SES, socioeconomic status.

* *p* < 0.05

** *p* < 0.01

*** *p* < 0.001.

The final model showed some remarkable similarities across the four examined groups. Specifically, parental perceptions that schools were providing opportunities for heritage culture and intercultural learning (multiculturalism) were positively related to all four outcomes of school‐based parental involvement (i.e., general school invitations, parent teacher relationship quality, school endorsement and parental involvement and volunteering) for all parents except for a nonsignificant relationship between multiculturalism and involvement and volunteering for minoritized parents in the United Kingdom (βs ≥ 0.19, *p*s < 0.001). Similarly, perceptions of a discriminatory school climate were negatively related to three out of four school involvement outcomes (i.e., endorsement, parent‐teacher relationship quality, and general school invitations) for all parents (βs ≤ −0.18, *p*s < 0.001). Interestingly, differences between countries and groups were found for assimilationism and its relationship to parental involvement measures. In the UK, the relationships were near zero and nonsignificant (βs ≤ 0.07, *p*s ≥ 0.183) across both parental groups. Unexpectedly, in Germany, perceived assimilationism was positively related to three outcomes (i.e., endorsement, parent‐teacher relationship quality, and general school invitations) for minoritized parents and one outcome for majoritized parents (endorsement; βs ≥ 0.15, *p*s ≤ 0.004). There was also an unexpected positive relationship between perceived discriminatory climate and volunteering among minoritized parents in Germany (β = 0.30, *p* <0.001).

To summarize, perceived multiculturalism of the school was positively related to parents' school involvement (for all involvement variables, supporting H3a), while perceptions of discriminatory practices were negatively related to parental school involvement except for the involvement and volunteering subscale (largely supporting H3c). Associations for assimilation were not as expected: it was not related to parental school involvement in the UK context, but positively related to three parental school involvement outcomes for minoritized parents (except involvement and volunteering) and one outcome for majoritized parents in Germany (failing to confirm H3b).

### 6.4 Exploratory Follow‐Up Analyses

To better understand the unexpected findings regarding the positive association between assimilationism and the parental involvement variables in minoritized parents in Germany, we conducted additional regression analyses that further distinguished the minoritized parents. The rationale behind these exploratory analyses was that we wanted to rule out that the minoritized parents in Germany differed from the minoritized parents in the United Kingdom in ways that accounted for the unexpected findings on assimilationism and involvement. Specifically, due to the aforementioned differences in sample ethnicity, minoritized parents in Germany could have experienced less discrimination or might be carrying more white privilege than the minoritized parent group in the UK. To investigate this idea, we considered the relationships between school diversity climate and parental involvement in the Muslim parents in Germany^4^ (*n* = 97), as this is a group that is known to experience substantial anti‐Muslim racism in Germany (e.g., German Centre for Integration and Migration Research DeZIM 2023) and therefore might feel more alienated in an assimilationist school climate. In this subgroup, assimilationism was not a significant predictor of any of the parental involvement variables (βs ≤ 0.16, *t*s ≤ 1.30 *p*s ≥ 0.19). The sign and strength of the effect was positive and small (between *β* = 0.12 and *β* = 0.16) for all parental involvement variables except involvement and volunteering (β = −0.12, *t* = −1.10 *p* = 0.27). Thus, the findings for the subsample are more in line with the findings for the UK sample and also violate our assumptions of a negative relationship with parental involvement.

## 7 Discussion

Our findings provide first evidence that parental perceptions of school diversity climate are shaped by national contexts and by experiences related to being part of ethnic and racially minoritized groups. As expected, perceptions of assimilationism and discrimination were higher in the national context with less well‐established multicultural policies and less stringent equality, diversity, and inclusion policies at the school level (Germany) than in the national context, where multiculturalism is more widely supported through policy and schools are required to formulate and implement concrete strategies in this area (UK). At the same time, and also in line with our hypotheses, minoritized parents perceived more assimilationism, less multiculturalism, and more discrimination in schools than majoritized parents. These findings complement and extend extant literature on school diversity climate (e.g., Baysu et al. 2023; Schachner et al. 2016), newly highlighting its importance for minoritized parents and its relationship to parental school involvement. These differences also align with previous research that describes how minoritized parents prepare and socialize their children (Umaña‐Taylor and Hill 2020) for discriminatory (school) realities (e.g., Glock et al. 2013; Kemper 2015; Moffitt et al. 2018).

Most importantly, though, our findings indicate that perceived school diversity approaches matter for parental school involvement. In line with expectations, for minoritized parents, perceptions of heritage culture and intercultural learning opportunities (multiculturalism) in school were positively related to parental school involvement, while perceptions of discriminatory practices were negatively related to parental school involvement for all variables except involvement and volunteering. These findings were also consistent and similar across minoritized and majoritized groups and national contexts, highlighting the importance of focusing on conditions in the schools to determine when parents feel welcome in schools and decide to center their educational activities on behaviors and relationships within the school (e.g., Hill 2022). They underline the value of creating school environments that discuss and include the cultures represented within them and actively oppose discriminatory behaviors and practices to foster good relationships and positive parental engagement with schools.

Some of our findings were unexpected, specifically with regard to the positive relationship between school assimilationism and parental school involvement (except the involvement and volunteering subscale) for minoritized parents in Germany. This finding is not easy to explain or interpret. Given the previous literature, it is unlikely that minoritized parents would want their cultures to be actively excluded from school practices. Instead, there might be an understanding fueled by integration needs and discourse (e.g., Gomolla and Kollender 2022) that the school, as a representative of the German state, has to take up the role of imparting “German culture” to some extent. Similarly, minoritized parents may perceive German society as assimilative and therefore feel that assimilation as a school practice better prepares their children for life in this society and is necessary for them to succeed (e.g., Phalet and Baysu 2020). That is, parents may have an (implicit) understanding that deviating from the schools' assimilation expectation could limit their children's chances of success in a system that frequently equates assimilation with achievement (Krachum Ott et al. 2025). This interpretation also ties in with the research mentioned above that describes some parents' in Germany actively seeking out more assimilative school contexts to promote their children's educational success and take on specific school tasks to demonstrate their belonging (Aral and Juang 2025). However, our additional analysis of the Muslim subsample in Germany suggests that the positive association between perceived assimilationism and school involvement may not hold for the most stigmatized minoritized groups. At the same time, assimilation did not show the expected negative relations with parental school‐based involvement in this particular group either, underlining the robustness of this overall finding.

In addition, the measurement of assimilation was questionable on two counts: first, the version in the United Kingdom referred to “British culture” as the overarching category, which is broader and more inclusive of ethnic and racially minoritized groups, and is discussed in this way in the UK context. “German culture,” in contrast, by connotation and common lay understanding, does not include a multicultural perspective. Second, the measurement of assimilationism was not as reliable in the UK sample as in the German sample, so that it is not possible to say whether this finding is indeed specific to the German context or not. Therefore, this finding should be interpreted with caution, and future research should further explore this unexpected result.

Finally, our results offer a more differentiated view of associations of school‐based parental involvement. In line with past research (e.g., Boonk et al. 2018; Kim et al. 2020), they suggest that it is important to consider both behavioral involvement and psychological engagement separately, as differential results emerge for the two aspects. This is illustrated in our findings regarding the involvement and volunteering in the school subscale, our most behavioral measure of parental involvement. First, the minoritized parents in this study reported higher levels of involvement and volunteering than majoritized parents, but there were no differences between these two groups on any of the other school‐based involvement subscales. Second, for all parents, self‐efficacy was highly and most strongly related to this measure, pointing to the importance of the relationship between behavior and efficacy. Thirdly, while the relationships between the climate measures and the other involvement variables were more consistent across groups and contexts, ‘involvement and volunteering’ was significantly predicted only by perceived multiculturalism, and in the expected direction (except for the minoritized group in Germany, where there was no relationship). It was not associated with perceived assimilationism or school discriminatory climate in either country—with one exception: in Germany, there was an unexpected positive association between perceived discriminatory climate and volunteering among minoritized parents. Further exploratory analyses with the individual items of the volunteering scale as dependent variables in the same regression analyses indicated that this unexpected relationship indicated an increased effort to provide appropriate materials to the schools. This shows the complex possible reactions to discrimination that can include disengagement or increased engagement of parents wanting to help and protect their children.

### 7.1 Limitations and Future Directions

Despite the novelty and interest of our findings, there are also some notable limitations to our work. First and foremost, we made a conscious decision to group together parents from many different ethnic, racial, and religious backgrounds into an overarching group of ethnic and racially minoritized parents. While this decision makes sense in light of our interest in generalizable processes, it could be criticized for adding to the literature that implies ethnic and racially minoritized parents form a kind of uniform group that is separate from parents of majoritized groups (e.g., Isik‐Ercan 2018). We do not intend to suggest this and fully acknowledge that the lived realities of different groups, including their experiences of marginalization, differ largely. For instance, in Germany, Black individuals and Muslims in particular face higher levels of discrimination than people with other backgrounds (German Centre for Integration and Migration Research DeZIM 2023). However, we still believe that our approach is justified by our conscious focus on institutional conditions of parental involvement, not familial conditions.

Second, and relatedly, despite the overall heterogeneity of the minoritized parents in our sample, there are some differences between the UK sample and the German sample, which might have affected our ability to draw comparisons between the two samples, especially had we found differences that applied only to the minoritized groups in each context (which we did not). The flipside of this argument is that this diversity actually increases the generalizability of our findings regarding the experience of belonging to a minoritized group, because our results are so similar across both contexts. This suggests that certain institutional dynamics may operate consistently across contexts. Nonetheless, future research should try to replicate our findings in other contexts, using samples that are more closely matched in terms of demography, ethnicity, and migration history: this will increase the interpretation of processes that are specific to particular ethnic and racially minoritized groups and those that are generalizable across groups or contexts (Benbow and Aumann 2020; Benbow et al. 2021). This would also provide a more precise understanding of how specific minoritized groups experience education systems and the unique structural barriers they face.

A third point regarding our sample is that it is limited by the fact that our participants were recruited through convenience sampling, which may introduce bias into our sample and makes it difficult to speak to the representativity of our sample and to rule out specific selection effects limiting the generalizability of our results. Research indicates that individuals from the most disadvantaged ethnic and racial groups are often less likely to participate in studies, particularly when recruitment relies on commercial methods that may not reach these groups effectively. Barriers such as digital access, mistrust of research, and socioeconomic constraints may prevent these populations from being included, leading to a potential skew in our sample (Passmore et al. 2022). Our sample had a high proportion of minoritized parents who were born in their country of residence (75% of the sample), and minoritized parents in our study also reported higher SES than would be expected in the general population. These specifics may partially explain why we found almost no differences in terms of mean levels of involvement between minoritized and majoritized parents, unlike the reviewed literature that has focused on barriers to involvement for minoritized parents. These sample characteristics may also have contributed to our results regarding assimilation and involvement for minoritized parents in Germany. We tried to counter some of these sampling effects by introducing quality checks and applying them to screen out cases. However, we would welcome future work that can replicate our findings using more robust and representative sampling procedures. Based on such sampling, future research could also extend our focus to consider the role and intersection of different social positioning variables, such as social class and gender, and their intersections with ethnic and racially minoritized status.

There were also some measurement issues in our study that should not be ignored: first, we had low internal consistency both for volunteering (in the overall sample) and for assimilationism (in the UK sample only). These were also the scales that needed adjustment to achieve partial scalar invariance. Thus, our results regarding these variables should be interpreted with particular caution and should be replicated with more reliable measures in future studies. Similarly, we would like to see more work that extends our findings to other measures of parental involvement, which may be more desirable and culturally appropriate for specific minoritized families (e.g., Antony‐Newman 2018; Hill 2022). Finally, our results are, of course, cross‐sectional in nature and therefore cannot speak to causality or process in the relationships we found—we would hope, however, that our results can be a starting point for future longitudinal studies, which ideally should extend to other institutional conditions. We would also like to see research that explicitly addresses the possibility of matches and mismatches between parental and institutional perspectives and considers how they differentially affect parental involvement.

## 8 Conclusions

Despite these limitations, this study provides first evidence that school diversity approaches matter not only for ethnic and racially minoritized students, but also for their parents and, specifically, for their involvement in school. Our results indicate that an overemphasis on familial conditions to explain parental involvement overlooks the critical role that school environments play, which is essential for understanding the complex dynamics of engagement. Our findings further suggest that it is important to consider school conditions in addition to familial conditions when asking why or whether parents get involved with schools, and that the effects of these may vary according to wider national policies that are in place, as suggested by the differential findings in the two examined contexts. Importantly, when asking why or whether parents get involved with schools the parents themselves should be the ones providing answers to these questions. Schools wanting to increase their accessibility to minoritized families within their community may benefit from explicitly evaluating, refining and communicating their approaches to diversity. In particular, our findings suggest that schools should both endorse and integrate heritage and cultural practices and ensure that antidiscrimination measures are effectively in place. These efforts are likely to support greater parental involvement not only among minoritized parents but also among majoritized parents, fostering more inclusive school communities overall. Ultimately, such practices may promote minoritized pupils' school adaptation and reduce structural inequities in education.

## Author Contributions

**Alison E. F. Benbow:** writing – original draft, writing – review and editing, methodology, data curation, formal analysis. **Gülseli Baysu:** conceptualization, funding acquisition, methodology, data curation, investigation, writing – review and editing. **Priscilla Krachum Ott:** writing – review and editing. **Maja K. Schachner:** conceptualization, funding acquisition, investigation, methodology, writing – review and editing. **Aileen Edele:** conceptualization, methodology, funding acquisition, investigation, writing – review and editing, supervision.

## Conflicts of Interest

The authors declare no conflicts of interest.

## Data Availability

The data that support the findings of this study are available at https://osf.io/j568k/overview?view_on and on request from the corresponding author. Demographic data are not publicly available due to privacy or ethical restrictions.

## Footnotes

1 In referring to ethnically and racially minoritized and majoritized groups we highlight that people differ in terms of their social positions shaped by processes of marginalization and privilege relating to race and ethnicity. We acknowledge that these groups are not monolithic; rather, how individuals' experience being part of these groups (and therefore holding these social positions) depends on their specific ethnic and racial positioning in broader society and in the school context.

2 In the UK, participants were asked to self‐identify using the categories White, Mixed or Multiple ethnic groups, Asian or Asian British, Black, African, Caribbean or Black British, and Other ethnic group. There was no comparative question in the German sample, so that we had to approximate this information from the nationalities of participants, and their parents. Therefore, this statement compares people self‐identifying as Asian or Asian British, Black, African, Caribbean or Black British in the UK sample with all participants from countries in Asia, Africa and Middle/South America in the German sample.

3 This finding was unexpected, because minoritized families in Germany and the United Kingdom have lower SES on average than majoritized families (e. g., Mirza and Warwick 2024; Schotte et al. 2025). To rule out the possibility that the finding was solely an artifact of the self‐report SES‐indicator in our study, we also examined participant's highest level of education. Chi‐squared tests indicated a significant overrepresentation of university degrees in our minoritized samples compared to the majoritized samples, who were overrepresented in the group with high school diplomas (*p*s < 0.001), thus confirming our finding regarding minoritized families' higher SES.

4 It was not possible to run a comparative analysis across groups, because there were not enough Muslim participants in each group.
